# Essential oil from *Lavandula angustifolia* elicits expression of three *SbWRKY* transcription factors and defense-related genes against sorghum damping-off

**DOI:** 10.1038/s41598-022-04903-x

**Published:** 2022-01-17

**Authors:** Younes M. Rashad, Elsayed S. Abdel Razik, Doaa B. Darwish

**Affiliations:** 1 Plant Protection and Biomolecular Diagnosis Department, Arid Lands Cultivation Research Institute, City of Scientific Research and Technological Applications (SRTA-City), New Borg El-Arab City, 21934 Egypt; 2 Botany Department, Faculty of Science, Mansoura University, Mansoura, Egypt

**Keywords:** Microbiology, Plant sciences

## Abstract

Sorghum damping-off, caused by *Fusarium solani* (Mart.) Sacc., is a serious disease which causes economic loss in sorghum production. In this study, antagonistic activity of lavender essential oil (EO) at 0.5, 0.75, 1.0, 1.25, 1.5, and 1.6% against *F. solani* was studied in vitro. Their effects on regulation of three *SbWRKY* transcription factors, the response factor *JERF3* and eight defense-related genes, which mediate different signaling pathways, in sorghum were investigated. Effects of application under greenhouse conditions were also evaluated. The results showed that lavender EO possesses potent antifungal activity against *F. solani*. A complete inhibition in the fungal growth was recorded for lavender EO at 1.6%. Gas chromatography–mass spectrometric analysis revealed that EO antifungal activity is most likely attributed to linalyl anthranilate, α-terpineol, eucalyptol, α-Pinene, and limonene. Observations using transmission electron microscopy revealed many abnormalities in the ultrastructures of the fungal mycelium as a response to treating with lavender EO, indicating that multi-mechanisms contributed to their antagonistic behavior. Results obtained from Real-time PCR investigations demonstrated that the genes studied were overexpressed, to varying extents in response to lavender EO. However, *SbWRKY1* was the highest differentially expressed gene followed by *JERF3*, which suggest they play primary role(s) in synchronously organizing the transcription-regulatory-networks enhancing the plant resistance. Under greenhouse conditions, treating of sorghum grains with lavender EO at 1.5% prior to infection significantly reduced disease severity. Moreover, the growth parameters evaluated, the activities of antioxidant enzymes, and total phenolic and flavonoid contents were all enhanced. In contrast, lipid peroxidation was highly reduced. Results obtained from this study support the possibility of using lavender EO for control of sorghum damping-off. However, field evaluation is highly needed prior to any usage recommendation.

## Introduction

Sorghum (*Sorghum bicolor* (L.) Moench) is among the most important cereal crops worldwide as a source of food/feed, and ethanol production. It is ranked the fifth main grain crop with a total global production of more than 59 million tons^1^.

Sorghum damping-off, caused by *Fusarium solani* (Mart.) Sacc., is a serious disease which causes seeds and seedling decay resulting in a significant economic loss in the crop yield^2^. In addition, *F. solani* is a toxigenic fungus which produces dangerous mycotoxins such as trichothecenes and fusaric acid affecting human and animal health^3,4^. The symptoms include seed decay in the soil, discoloration and rotting of the radicles which prevent germination and emergence, and formation of red lesions on the roots of seedlings that do emerge, which, especially at low temperatures, halts their development^5^. Various chemical fungicides are available for control of damping-off disease such as Mancozeb, Rizolex, and Benomyl^6^, but the use of chemical fungicides is unfavorable owing to potential deleterious health effects and environmental risks^7^.

Essential oils (EOs) and plant extracts have been extensively studied by many researchers as alternatives to chemical fungicides due to their ecological safety, and potent antifungal activities against several phytopathogenic fungi^8–10^. In this regard, Ghoneem et al.^11^ reported full suppression in the fungal growth of *Sclerotinia sclerotiorum* by clove essential oil at 2%. The rich content of different bioactive components in EOs such as phenols, coumarins, quinines, flavonoids, tannins, and fatty acids provides multifunctional and synergistic antifungal potentialities against plant pathogenic fungi. In addition, these multifunctional bioactive compounds make development of microbial-resistance difficult based on diverse antagonistic modes of action^9,12^. Moreover, EOs of some medicinal plants may act as elicitors, triggering the plant defense-responses against attacking pathogens^8,13^.

WRKY proteins represent a pivotal plant family of transcription factors (TF) which work via interconnected signaling networks to synchronously regulate a diverse set of defense-responses against biotic and abiotic stresses, as well as metabolic responses^14^. Many investigations have implicated *WRKY* TFs in regulation of defense-responses against different fungal diseases^15,16^. Overexpression of *OsWRKY45* in rice provides resistance against the blast fungus (*Magnaporthe oryzae*) via triggering salicylic acid (SA)-signaling pathway genes^17^. Also, overexpression of *VvWRKY1* in grapevine elicits expression of jasmonic acid (JA)-signaling pathway genes against the downy mildew fungus (*Plasmopara viticola*)^18^. Recently, ninety four *SbWRKY* TFs were identified in sorghum and classified into three groups according to their binding domains and type of zinc-finger motifs^19^.

*Lavandula angustifolia* Mill., frequently known as English lavender, is a flowering shrub which belongs to family Lamiaceae. It has many uses, such as flavoring agent in foods, pharmaceutical uses such as soap, perfumes and cosmetics manufactures, as well as many therapeutic applications owing to its antimicrobial, antioxidant, anxiolytic, antispasmodic, and aphrodisiac properties^20^. Antifungal activity of lavender EO has been studied against different pathogenic fungi. Xiong et al*.*^21^ reported a complete fungal inhibition for Lavender EO at 800 μL/L against *Monilinia fructicola.* Their study indicated that the utilized antagonistic mechanisms included destruction of the cell membrane, cytoplasmic leakage, and induction of cell apoptosis. In addition, the antifungal activity of lavender EO was reported also against *Candida albicans*^22^. The present study aimed to (1) investigate the antagonistic activity of lavender EO against *F. solani* in vitro, especially effects on ultrastructures, (2) study their effect(s) on regulation of three *SbWRKY* TFs (1, 19, and 45), Jasmonate and ethylene-response factor 3 (*JERF3*) and eight defense-related genes, which mediate SA, JA and ethylene (ET)-signaling pathways, in sorghum against *Fusarium* damping-off, (3) evaluate their biocontrol activity under greenhouse conditions, as well as their effects on the growth and biochemical plant parameters.

## Results

### Screening for antifungal activity of lavender EO in vitro

**Figure 1:**
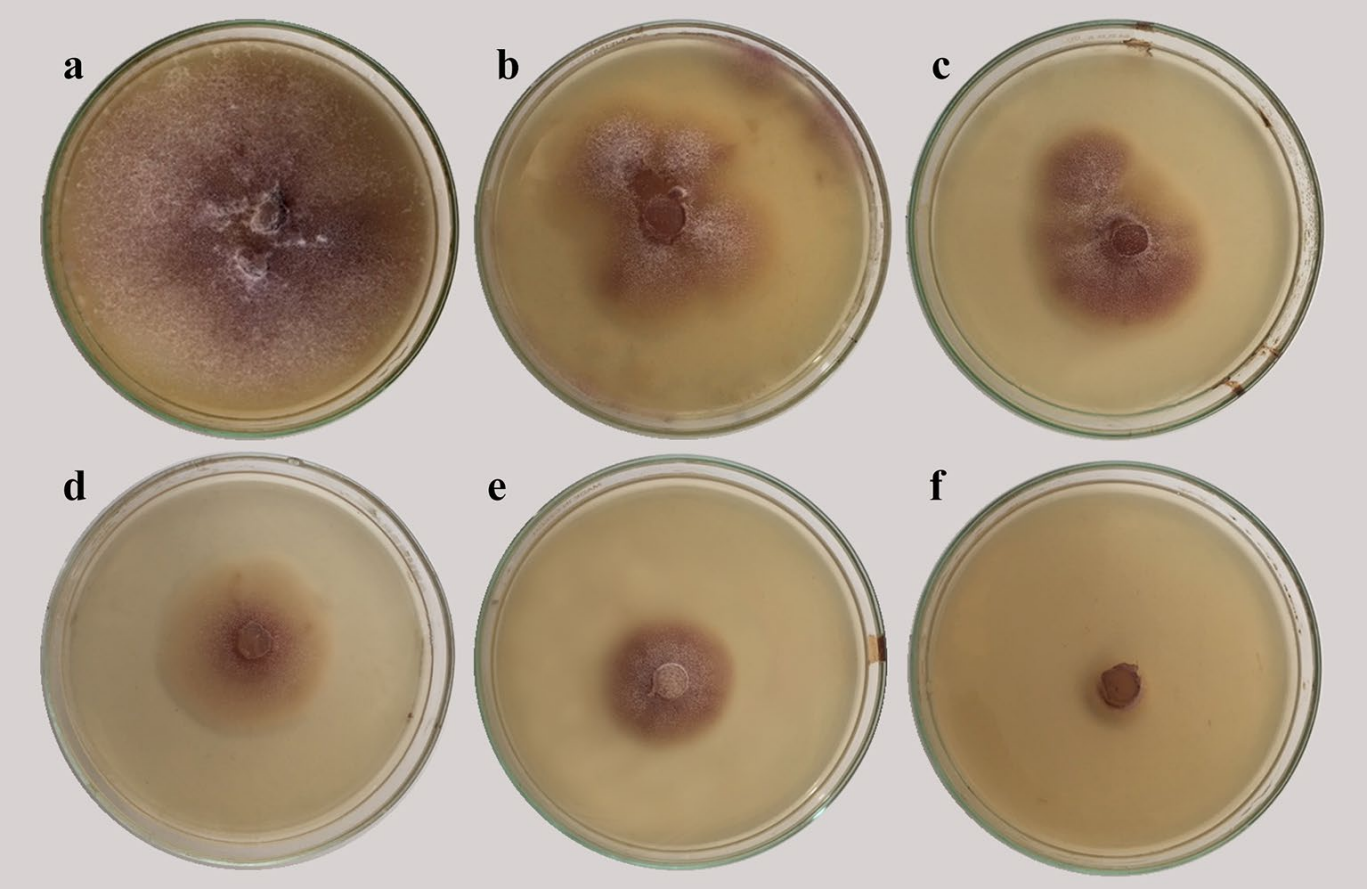
Antifungal activity of lavender essential oil at different concentrations against *Fusarium solani* in vitro, where a: 0, b: 0.5, c: 0.75, d: 1, e: 1.25, and f: 1.5 %.

Antifungal activity of lavender EO was assessed in vitro against *F. solani* at 0, 0.5, 0.75, 1, 1.25, 1.5 and 1.6% (Fig. 1). The mean reductions in the fungal growth are presented in Table 1. All tested concentrations exhibited inhibitory effects in varying extents compared with the control treatment. Growth inhibition was elevated with the increment in the concentration of lavender EO. The highest growth inhibition (97.6%) was obtained at 1.5% recording 2 mm radial growth compared with the control and the lowest growth inhibition was recorded at the concentration of 0.5%. The minimum inhibitory concentration (MIC) was obtained at 1.6%, while 50% inhibitory concentration (IC50) was recorded at 0.65%.

**Table 1:** Growth inhibition (%) of *Fusarium solani* when exposed to lavender essential oil at different concentrations.

Treatment	Radial growth (mm)	Growth inhibition (%)
Control	83.0 ± 1.2^a^	0.0^f^
Chemical fungicide	00	100
**Lavender EO (%)**		
0.5	49.0 ± 2.1^b^	41.0^e^
0.75	36.4 ± 1.9^c^	56.1^d^
1	28.0 ± 1.6^d^	66.3^c^
1.25	16.0 ± 0.9^e^	80.7^b^
1.5	2.0 ± 0.6^f^	97.6^a^
1.6	00	100

Values followed by the same letter are not significantly different according to Duncan’s multiple range test (*P* ≤ 0.05), each value represents the mean of 3 replicates ± SD. Chemical fungicide = nystatin at 50 µg/mL.

### Scanning electron microcopy observations (SEM)

**Figure 2:**
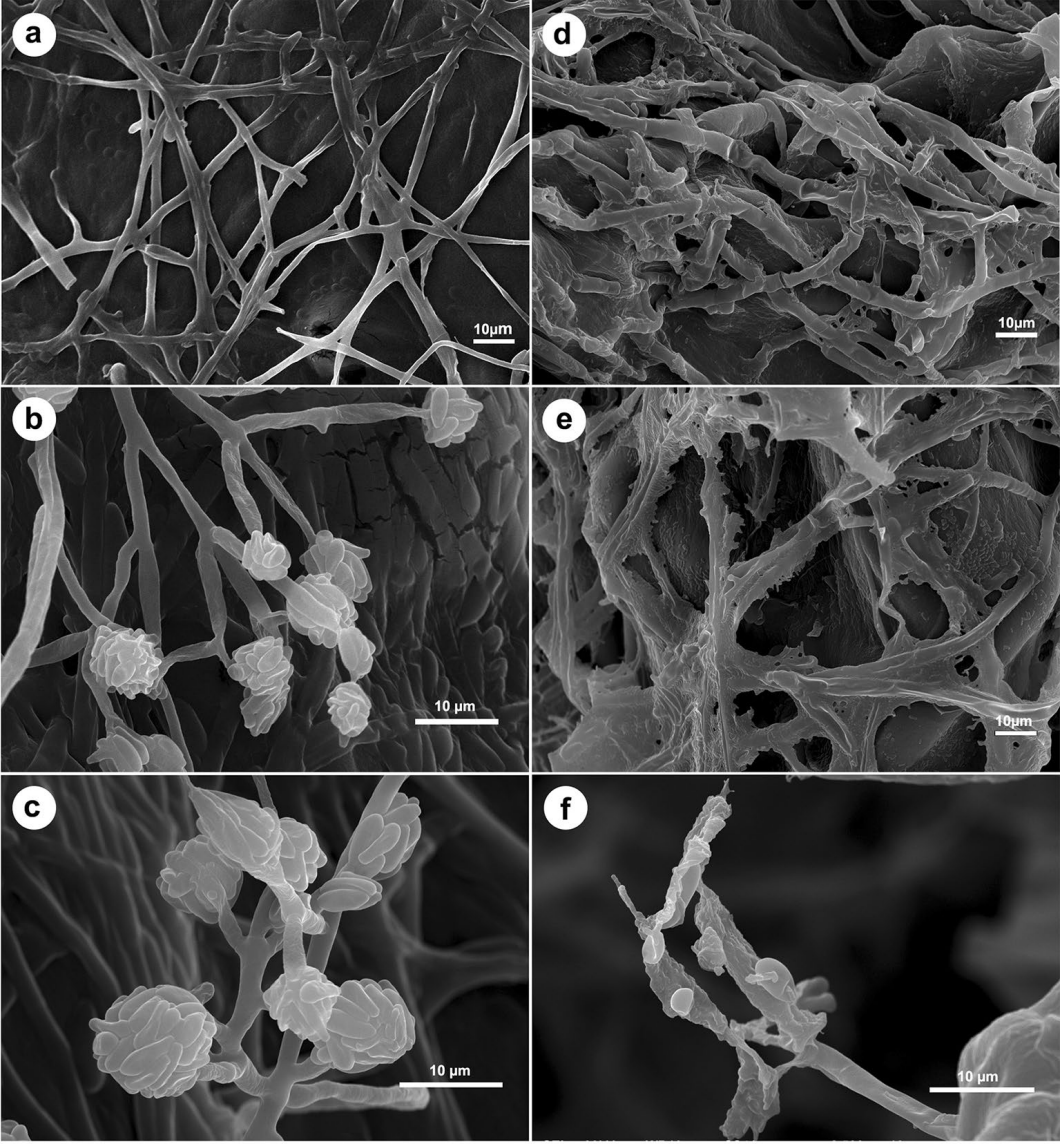
Scanning electron micrographs showing effects of lavender essential oil on morphology of *Fusarium solani.* The untreated fungus shows normal, intact, thick, and regular mycelia with smooth surface (**a**), typical, long, unbranched, and smooth-surfaced aerial conidiophores bearing false heads of ellipsoidal, non-septated microconidia (**b** and **c**). Mycelia of *F. solani* treated with lavender EO show severe collapse, distortion, shrinking, squashing, deformation, and rough surface with no observed conidia (**d**–**f**).

The antifungal effects of lavender EO on morphology of *F. solani* were examined using SEM to investigate its antagonistic mechanisms. SEM observations of the untreated fungus showed normal, intact, thick, and regular mycelia with smooth surface (Fig. 2a). In addition, typical, long, unbranched, and smooth-surfaced aerial conidiophores bearing false heads of ellipsoidal, non-septated microconidia were also observed (Fig. 2b,c). In contrast, mycelia of *F. solani* treated with lavender EO showed dramatic alterations in their morphology including severe collapse, distortion, shrinking, squashing, deformation, and rough surface. Furthermore, no conidia were detected in the treated fungal mycelia (Fig. 2d–f).

### Transmission electron microcopy observations (TEM)

**Figure 3:**
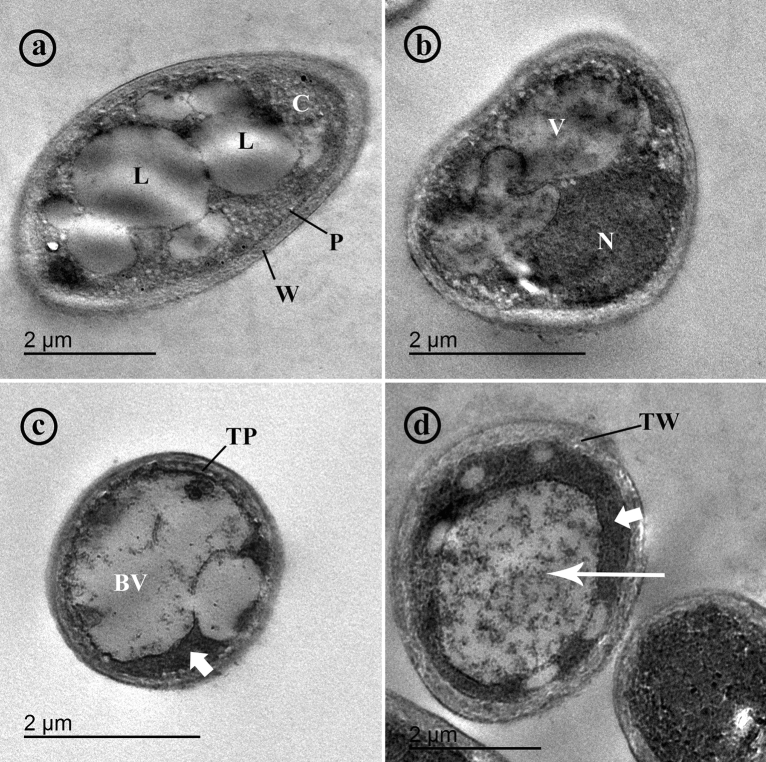
Transmission electron micrographs of a cross section in hyphae of *Fusarium solani*. Where, (**a**) and (**b**) show untreated hyphae (control). Note cell wall (W), plasmalemma (P), cytoplasm (C), lipid globules (L), nucleus (N) and vacuoles (V), while, (**c**) and (**d**) show hyphae treated with lavender essential oil at 1.25%. Note a thick plasmalemma (TP), big vacuoles (BV), electron-dense cytoplasm (short arrows), thick wall (TW) and vacuoles contain electron-dense materials (long arrow).

Observation of untreated (control) hyphae of *F. solani* using TEM exhibited normal ultrastructure. Thin cell walls and plasmalemma embracing the cytoplasm with electron-lucent lipid globules, nucleus, and vacuoles were noted (Fig. 3a,b). In contrast, TEM observations of *F. solani* hyphae treated with lavender EO showed considerable ultrastructural alterations. Thick cell walls and plasmalemma enclosing an electron-dense cytoplasm were observed. Large vacuoles containing electron-dense materials and absence of the lipid globules were also noted (Fig. 3c,d).

### Gas chromatography–mass spectrometry (GC–MS)

**Figure 4:**
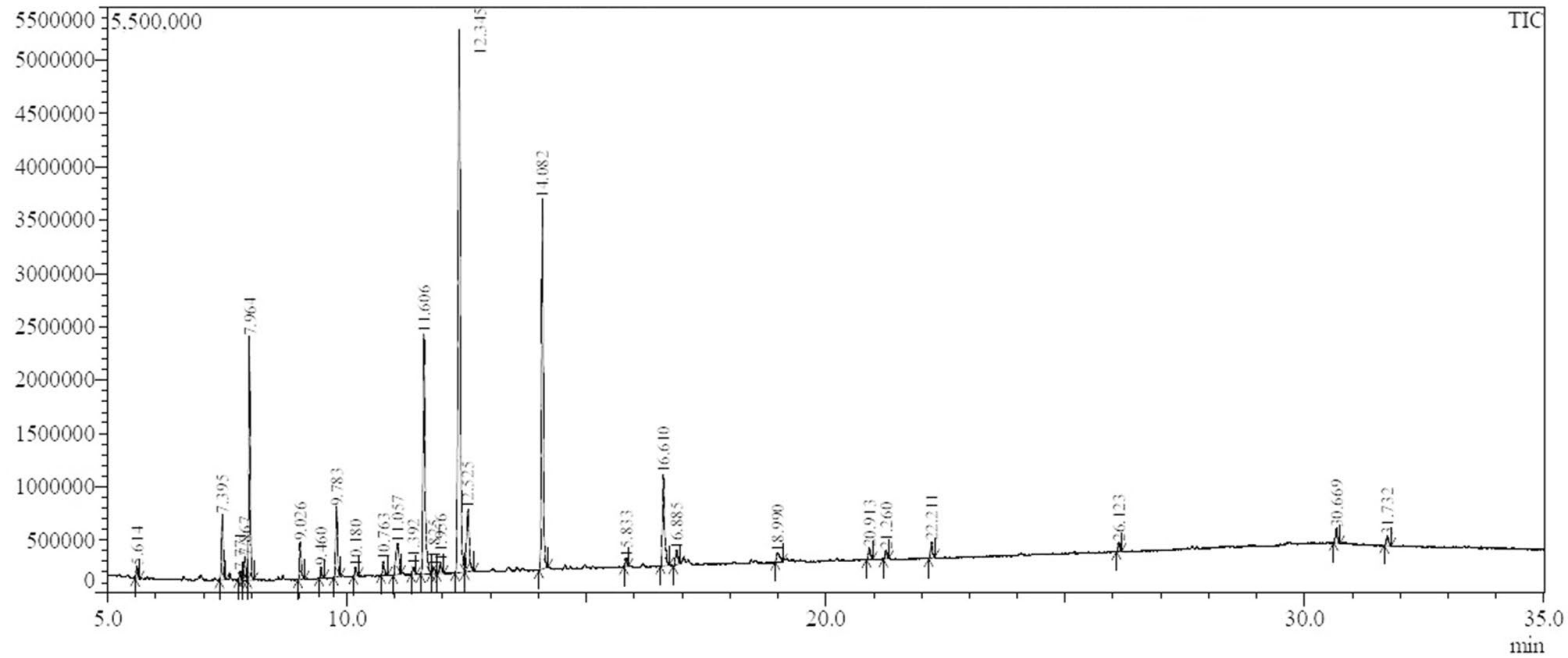
GC–MS chromatogram showing the chemical composition of lavender essential oil.

The chemical composition of lavender EO was analyzed via GC–MS (Fig. 4). Twenty-eight compounds in varying proportions were identified (Table 2). The major components in lavender EO included; linalool (31.1%), linalyl anthranilate (16.8%), benzyl acetate (12.9%), and 1,8-cineole (10.1%). Other components were identified in intermediate proportions including α-Terpineol acetate (4.92%), ɣ-Terpineol (3.89%), α-Terpineol (3.15%), α-Pinene (2.85%), Dihydrocarveol (2.34%), Dihydromyrcenol (1.81%), and Limonene (1.04%), while, the rest components were present in minor ratios.

**Table 2:** Chemical composition of lavender essential oil using GC–MS system.

Peak #	Retention time (min)	Peak area (%)	Compound name
1	5.614	0.52	3-Carene
2	7.395	2.85	α-Pinene
3	7.771	0.26	p-Cymene
4	7.867	1.04	Limonene
5	7.964	10.1	1,8-Cineole
6	9.026	1.81	Dihydromyrcenol
7	9.460	0.52	α-Terpinolen
8	9.783	3.15	α-Terpineol
9	10.180	0.26	Fenchol
10	10.763	0.78	1-Terpinenol
11	11.057	2.34	Dihydrocarveol
12	11.392	0.26	Isoborneol
13	11.606	12.9	Benzyl acetate
14	11.825	0.26	3,5,5-Trimethylhexyl acetate
15	11.956	0.52	Terpinen-4-ol
16	12.345	31.1	Linalool
17	12.525	3.89	ɣ-Terpineol
18	14.082	16.8	Linalyl anthranilate
19	15.833	0.26	p-Cymen-8-ol
20	16.610	4.92	α-Terpineol acetate
21	16.885	0.78	Eugenol
22	18.990	0.78	Geranyl acetate
23	20.913	0.78	Indan-1,3-diol monopropionate
24	21.260	0.52	Isoamyl salicylate
25	22.211	0.78	Amyl salicylate
26	26.123	0.52	α-Hexylcinnamaldehyde
27	30.669	0.78	Lilial
28	31.732	0.52	Astratone

### Transcript levels of three *SbWRKY* TFs and nine defense-related genes

**Figure 5:**
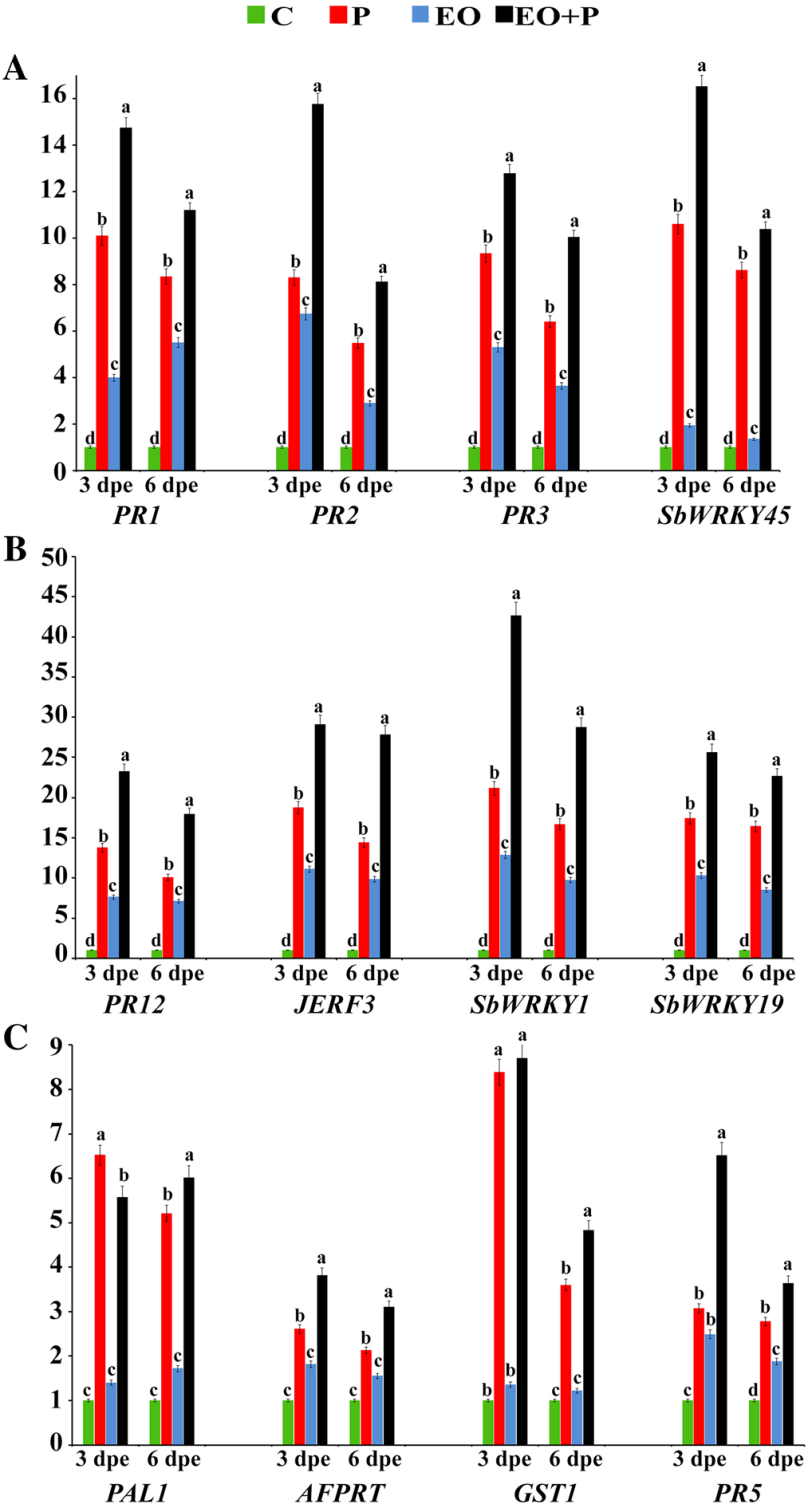
Histograms showing relative transcriptional expression levels of three *SbWRKY* transcription factors and some defense-related genes in sorghum plants infected with *Fusarium solani* and/or treated with lavender essential oil at 1.5% after 3 and 6 days post-emergence (dpe). C: untreated control, P: infected with *F. solani*, EO: treated with lavender essential oil at 1.5%, and P + EO: infected with *F. solani* and treated with lavender essential oil at 1.5%. In each time for each studied gene, columns superscripted with the same letter are not significantly different according to Duncan’s multiple range test (*P* ≤ 0.05). Each value represents the mean of three biological replicates; each sample was analyzed in triplicate. Error bars represent standard errors.

Transcriptional expression profiles of three *SbWRKY* TFs, *JERF3* and eight defense-related genes in sorghum shoot were studied 3 and 6 days post emergence (dpe) (Fig. 5). In every case, EO in the presence of the pathogen increased the level of expressions as measured by mRNA levels and uninoculated controls had the least mRNA. Inoculated plants in the absence of EO were also higher than controls and in most but not all cases expression induced by EO alone was also above the control level. Of all studied genes, *SbWRKY1* was the highest expressed gene followed by *JERF3*. For *SbWRKY1* expression, infection of sorghum plants with *F. solani* or treating with lavender EO induced their transcript level, but the transcriptional expression in the infected plants was much higher (21-fold at 3 dpe) than in the EO treated-plants when compared with the untreated control plants. However, the highest expression level was recorded for the infected plants also treated with lavender EO (43-fold at 3 dpe). For all treatments, the expression level of *SbWRKY1* at 6 dpe was lower than that at 3 dpe. The expression level of *JERF3* came in second after *SbWRKY1* and was triggered by infection with *F. solani* and/or treating with lavender EO, compared with the untreated control plants, but the expression level of the dual treatment was higher than the single treatments recording 29- and 28-fold at 3 and 6 dpe, respectively. Concerning *PR1*, *PR2*, *PR3*, *PR5*, *PR12*, *SbWRKY19* and *SbWRKY45*, infection with *F. solani* and/or treating with lavender EO induced the gene expression level at 3 and 6 dpe in varying degrees. Regarding *PAL1*, *AFPRT*, and *GST1*, the untreated-infected sorghum plants or infected plants which were treated with EO showed considerable up-regulation in the transcript level of the three genes, but the dual treatment was more highly induced than the infection-alone treatment. In contrast, sorghum plants treated with lavender EO did not exhibit any significant difference in the expression level of these three genes, when compared with the untreated control plants. In all expression profiles, the transcript level of the studied genes reduced from 3 to 6 dpe.

### Hierarchical clustering analysis

**Figure 6:**
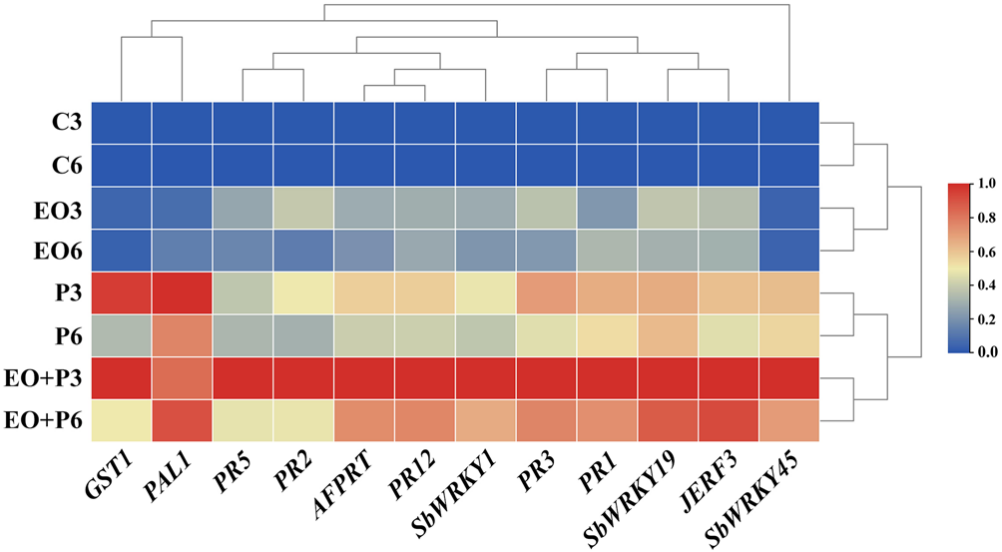
Hierarchical clustering heat map of transcriptional expression of three *SbWRKY* transcription factors and some defense-related genes in sorghum plant infected with *Fusarium solani* and/or treated with lavender essential oil at 1.5% after 3 and 6 days post-emergence (dpe). Where, C: untreated control, P: infected with *F. solani*, EO: treated with lavender essential oil at 1.5%, and P + EO: infected with *F. solani* and treated with lavender essential oil at 1.5%.

Hierarchical clustering heat map of transcriptional expression of the investigated genes in sorghum shoot is illustrated in Fig. 6. As seen from the heat map, all tested treatments are grouped into two main clusters, the first represents the untreated control plants, and the lavender-EO-treated plants at 3 and 6 dpe, while the other represents the infected plants whether treated with lavender EO or not at 3 and 6 dpe. In the first cluster, the untreated control plants at both investigated times (3 and 6 dpe) are grouped together in a separate subcluster, while, the lavender-EO-treated plants at the same times are grouped together in the other subcluster. In the second main cluster, the infected plants at the investigated times are grouped together in a separate subcluster, while, the infected plants which treated with lavender EO at 3 and 6 dpe are grouped together in another separate subcluster. Concerning the gene clustering, all genes are grouped into two main clusters, the transcription factor *SbWRKY45* is grouped in a separate out-cluster revealing its unique behavior, while, the other main cluster included all the other investigated genes. Moreover, the hierarchical clustering heat map shows 5 two-genes-clusters between *GST1*-*PAL1*, *PR5*-*PR2*, *AFPRT*-*PR12*, *PR3*-*PR1*, and *SbWRKY19*-*JERF3.* In general, the hierarchical clustering expression exhibited high up-regulation of the investigated genes in case of the infection treatments, whether treated with lavender EO or not. The maximum transcription levels were observed for the infected plants treated with lavender EO at 3 dpe.

### Disease assessment

Disease assessment data of the infected sorghum seedlings in response to treatment with lavender EO at different concentrations are presented in Table 3. The data indicated that the infection with *F. solani* caused damping-off of sorghum leading to up to 92% mortality, when compared with the untreated control treatment. Typical symptoms of *Fusarium* damping-off were recorded, including seed rotting, and pre- and post-emergence damping-off. In contrast, treating of sorghum grains with lavender EO prior to infection with *F. solani* led to a reduction in the disease severity, which increased with increased EO concentration. In this regard, the best result was recorded for the sorghum grains treated with lavender EO at 1.5% prior to the infection (17.7% mortality), which was essentially identical to treatment with the chemical fungicide.

**Table 3:** Disease assessment of sorghum seedlings infected with *Fusarium solani* in response to treating with lavender essential oil at different concentrations.

Treatment**	Seedling mortality (%)			Survival plants (%)
	Seed rot	Pre-emergence damping-off	Post-emergence damping-off	
C	3.0 ± 0.4^bc^	3.7 ± 0.6^d^	0.7 ± 0.05^c^	92.6 ± 2.1^c^
P	14.7 ± 1.2^a^	55.0 ± 4.8^a^	22.3 ± 3.3^a^	8.0 ± 1.0^f^
P + F	2.7 ± 0.3^c^	8.0 ± 0.9^c^	7.3 ± 1.3^b^	82.0 ± 1.7^d^
EO_1.0_	2.7 ± 0.5^c^	1.3 ± 0.3^de^	0.0^c^	96.0 ± 2.3^b^
EO_1.25_	0.7 ± 0.03^d^	0.0^e^	0.0^c^	99.3 ± 0.1^a^
EO_1.5_	0.0^d^	0.0^e^	0.0^c^	100 ± 0.0^a^
P + EO_1.0_	4.7 ± 0.5^b^	13.0 ± 1.7^b^	10.0 ± 1.5^b^	72.3 ± 1.1^e^
P + EO_1.25_	3.3 ± 0.6^bc^	9.0 ± 1.3^c^	8.3 ± 0.9^b^	79.4 ± 2.3^d^
P + EO_1.5_	3.0 ± 0.3^bc^	7.7 ± 1.1^c^	7.0 ± 0.6^b^	82.3 ± 3.8^d^

Values followed by the same letter are not significantly different according to Duncan’s multiple range test (*P* ≤ 0.05), each value represents the mean of 3 replicates ± SD.

**C: untreated control, P: infected with *F. solani*, P + F: infected with *F. solani* and treated with chemical fungicide (Rhizolex-T), EO_1.0_, EO_1.25_, EO_1.5_: treated with lavender EO at 1.0, 1.25, and 1.5% respectively, P + EO_1.0_, P + EO_1.25_, and P + EO_1.5_: infected with *F. solani* and treated with lavender EO at 1.0, 1.25, and 1.5%, respectively.

### Effect on plant growth

Results of the growth parameter evaluations obtained from the greenhouse experiment in response to treating with lavender EO at different concentrations and infection with *F. solani* are presented in Table 4. Infection of sorghum plants with *F. solani* led to a considerable reduction in growth, shoot weight and root weight at 30 and 45 dap, when compared with the untreated control plants. In contrast, treating of sorghum grains with lavender EO significantly enhanced the growth of sorghum plants compared with the untreated control plants. The growth promoting effect increased with the increment in EO concentration. The highest growth parameters were recorded for the sorghum plants treated with lavender EO at 1.5% harvested at both 30 and 45 dap. Compared to the treatment with the chemical fungicide, sorghum plants treated with lavender EO prior to infection with *F. solani* showed higher growth for plant height, shoot and root dry weights, than the untreated-infected sorghum plants. In this regard, the growth enhancing effect is directly proportional to the EO concentration at 30 and 45 dap.

**Table 4:** Growth parameters of sorghum plants infected with *Fusarium solani* in response to treating with lavender essential oil at different concentrations.

Treatment**	Plant height (cm)		Shoot dry weight (g)		Root dry weight (g)	
	30 dap	45 dap	30 dap	45 dap	30 dap	45 dap
C	18.8 ± 0.4^d^	30.6 ± 0.9^c^	0.14 ± 0.01^c^	0.21 ± 0.01^c^	0.11 ± 0.09^c^	0.14 ± 0.01^c^
P	4.1 ± 0.2^i^	11.7 ± 1.5^h^	0.04 ± 0.01^f^	0.07 ± 0.01^g^	0.03 ± 0.01^g^	0.06 ± 0.01^f^
P + F	16.3 ± 0.9^e^	28.9 ± 0.9^d^	0.12 ± 0.01^d^	0.14 ± 0.01^d^	0.09 ± 0.01^d^	0.12 ± 0.01^d^
EO_1.0_	20.7 ± 0.4^c^	30.3 ± 0.9^cd^	0.16 ± 0.02^b^	0.24 ± 0.02^b^	0.14 ± 0.02^b^	0.19 ± 0.01^b^
EO_1.25_	22.2 ± 0.3^b^	33.9 ± 1.2^b^	0.19 ± 0.02^a^	0.32 ± 0.02^a^	0.17 ± 0.04^a^	0.22 ± 0.01^a^
EO_1.5_	24.5 ± 0.7^a^	38.3 ± 1.4^a^	0.20 ± 0.02^a^	0.31 ± 0.01^a^	0.18 ± 0.05^a^	0.23 ± 0.01^a^
P + EO_1.0_	6.3 ± 0.1^h^	18.8 ± 0.4^g^	0.07 ± 0.01^e^	0.09 ± 0.01^f^	0.05 ± 0.01^ fg^	0.09 ± 0.01^e^
P + EO_1.25_	8.9 ± 0.3^g^	24.5 ± 0.6^f^	0.08 ± 0.01^e^	0.12 ± 0.01^e^	0.06 ± 0.01^ef^	0.10 ± 0.01^e^
P + EO_1.5_	11.4 ± 1.0^f^	27.4 ± 0.5^e^	0.08 ± 0.01^e^	0.14 ± 0.01^d^	0.07 ± 0.02^de^	0.11 ± 0.01^d^

Values followed by the same letter are not significantly different according to Duncan’s multiple range test (*P* ≤ 0.05), each value represents the mean of 3 replicates ± SD.

**C: untreated control, P: infected with *F. solani*, P + F: infected with *F. solani* and treated with chemical fungicide (Rhizolex-T), EO_1.0_, EO_1.25_, EO_1.5_: treated with lavender EO at 1.0, 1.25, and 1.5% respectively, P + EO_1.0_, P + EO_1.25_, P + EO_1.5_: infected with *F. solani* and treated with lavender EO at 1.0, 1.25, and 1.5%, respectively, and dap: days after planting.

### Effects on activities of antioxidant enzymes

Effects of lavender EO on activities of different antioxidant enzymes of sorghum plants infected with *F. solani* are shown in Table 5. Data obtained indicated that infection of sorghum plants with *F. solani* led to an induction in the activities of all studied enzymes when compared with the untreated control plants at 30 and 45 dap. In general, activity of catalase (CAT) and superoxide dismutase (SOD) enzymes in sorghum plants at 30 dap was higher than that at 45 dap, while, activity of ascorbate peroxidase (APX) and polyphenol oxidase (PPO) enzymes increased from 30 to 45 dap. Treating of sorghum plants with lavender EO at different concentrations significantly triggered activity of all tested enzymes compared with the untreated control. However, the inducing effect resulting from infection was more than that of the lavender EO treatments at both studied times. For all studied enzymes, the highest enzyme activity was recorded for the infected sorghum plants also treated with lavender EO at 1.5%, when compared with the treatment of the chemical fungicide. In this regard, the inducing effect on enzymes activity is directly proportional to the EO concentration at 30 and 45 dap.

**Table 5:** Activities of antioxidant enzymes in sorghum plants infected with *Fusarium solani* in response to treatment with lavender essential oil at different concentrations.

Treatment**	CAT (U min^−1^ mg^−1^ protein)		APX (U min^−1^ mg^−1^ protein)		SOD (U min^−1^ mg^−1^ protein)		PPO (U min^−1^ mg^−1^ protein)	
	30 dap	45 dap	30 dap	45 dap	30 dap	45 dap	30 dap	45 dap
C	47.6 ± 1.7^f^	46.5 ± 2.1^g^	12.7 ± 2.9^e^	14.3 ± 0.9^g^	36.3 ± 1.5^e^	35.7 ± 2.1^g^	8.5 ± 0.4^f^	11.1 ± 0.7^e^
P	56.2 ± 2.9^d^	53.7 ± 3.1^c^	23.7 ± 2.1^b^	24.0 ± 1.1^c^	56.1 ± 2.9^b^	47.6 ± 2.0^d^	18.5 ± 1.2^b^	20.4 ± 1.0^a^
P + F	56.4 ± 1.3^d^	51.3 ± 1.9^d^	23.6 ± 2.2^b^	23.4 ± 1.9^ cd^	55.6 ± 3.8^c^	46.1 ± 1.7^e^	17.8 ± 1.0^c^	18.2 ± 0.4^b^
EO_1.0_	48.1 ± 2.5^e^	47.2 ± 1.4^f^	18.2 ± 1.6^d^	19.2 ± 2.0^f^	36.5 ± 2.2^e^	35.8 ± 2.0^g^	9.3 ± 0.3^e^	10.8 ± 0.5^e^
EO_1.25_	48.6 ± 1.7^e^	47.3 ± 2.5^f^	19.3 ± 1.4^c^	21.5 ± 5.1^e^	37.6 ± 1.7^d^	36.7 ± 1.3^ fg^	10.2 ± 0.4^d^	12.1 ± 0.8^d^
EO_1.5_	48.7 ± 1.9^e^	48.2 ± 1.1^e^	19.5 ± 1.3^c^	21.9 ± 1.3^e^	38.1 ± 1.2^ cd^	37.5 ± 2.6^f^	10.4 ± 0.5^d^	13.3 ± 0.9^c^
P + EO_1.0_	59.7 ± 1.9^c^	53.7 ± 2.5^c^	23.3 ± 2.5^b^	26.6 ± 2.2^b^	56.2 ± 1.5^b^	48.1 ± 1.3^c^	17.9 ± 0.8^c^	18.8 ± 1.3^b^
P + EO_1.25_	61.2 ± 2.2^b^	54.6 ± 2.6^b^	23.6 ± 2.1^b^	26.2 ± 1.7^b^	58.3 ± 1.1^a^	49.4 ± 1.2^b^	19.5 ± 0.5^a^	20.4 ± 1.1^a^
P + EO_1.5_	65.3 ± 2.7^a^	60.2 ± 2.4^a^	25.2 ± 1.9^a^	27.8 ± 1.2^a^	58.7 ± 2.2^a^	50.8 ± 1.1^a^	19.7 ± 0.3^a^	20.9 ± 1.6^a^

Values followed by the same letter are not significantly different according to Duncan’s multiple range test (*P* ≤ 0.05), each value represents the mean of 3 replicates ± SD.

**C: untreated control, P: infected with *F. solani*, P + F: infected with *F. solani* and treated with chemical fungicide (Rhizolex-T), EO_1.0_, EO_1.25_, EO_1.5_: treated with lavender EO at 1.0, 1.25, and 1.5% respectively, P + EO_1.0_, P + EO_1.25_, and P + EO_1.5_: infected with *F. solani* and treated with lavender EO at 1.0, 1.25, and 1.5%, respectively. CAT: catalase enzyme, APX: ascorbate peroxidase enzyme, SOD: superoxide dismutase enzyme, PPO: polyphenol oxidase enzyme, and dap: days after planting.

### Effects on lipid peroxidation, total phenolic and flavonoid contents

Effects of lavender EO on lipid peroxidation, total phenolic and flavonoid contents of sorghum plants infected with *F. solani* are presented in Table 6. Results of biochemical analyses of sorghum plants showed that infection with *F. solani* led to significant elevations in lipid peroxidation, total phenolics and flavonoids at 30 and 45 dap, when compared with the untreated control plants. In contrast, treating with lavender EO at different concentrations did not affect lipid peroxidation of sorghum plants. Treating of the infected sorghum plants with lavender EO significantly reduced the lipid peroxidation, compared with the treatment of the chemical fungicide. This effect is directly proportional to the EO concentration at 30 and 45 dap. The lipid peroxidation in sorghum plants at 30 dap was higher than that at 45 dap. Regarding total phenolic and flavonoid contents, the data showed that treating of sorghum plants with lavender EO at different concentrations significantly increased both categories of compounds in direct proportional relationship at 30 and 45 dap. The highest levels of phenolics and flavonoids were recorded for the infected sorghum plants treated with lavender EO at 1.5%, compared with the chemical fungicide treatment at 30 and 45 dap. In general, levels of both increased from 30 to 45 dap in all sorghum plants treatments.

**Table 6:** Lipid peroxidation, total phenolic and flavonoid contents in sorghum plants infected with *Fusarium solani* in response to treatment with lavender essential oil at different concentrations.

Treatment**	MDA (nmol g^−1^ dry wt)		TPC (mg catechol 100 g^−1^ dry wt)		TFC (mg rutin 100 g^−1^ dry wt)	
	30 dap	45 dap	30 dap	45 dap	30 dap	45 dap
C	11.7 ± 0.6^g^	9.1 ± 0.9^f^	76.2 ± 1.4^h^	98.2 ± 1.4^g^	38.2 ± 1.5^h^	49.2 ± 1.1^g^
P	43.5 ± 1.7^a^	42.4 ± 2.1^a^	219.4 ± 3.1^c^	232.4 ± 2.1^c^	191.3 ± 2.3^e^	211.3 ± 2.6^d^
P + F	18.7 ± 0.3^c^	17.8 ± 1.1^c^	167.4 ± 1.2^d^	188.3 ± 3.9^d^	200.4 ± 1.8^d^	213.4 ± 1.4^d^
EO_1.0_	11.3 ± 1.5^g^	9.7 ± 1.4^f^	116.4 ± 2.5^f^	137.5 ± 2.0^f^	76.3 ± 1.2^g^	81.8 ± 1.8^f^
EO_1.25_	11.4 ± 0.9^g^	9.9 ± 1.8^f^	118.3 ± 1.4^f^	142.3 ± 3.7^e^	78.8 ± 2.4^g^	83.6 ± 2.2^f^
EO_1.5_	10.3 ± 0.8^h^	9.1 ± 1.1^f^	122.6 ± 3.2^e^	144.2 ± 1.8^e^	91.6 ± 2.0^f^	102.3 ± 2.0^e^
P + EO_1.0_	22.8 ± 1.1^b^	19.3 ± 0.5^b^	233.2 ± 2.5^b^	245.4 ± 3.4^b^	212.6 ± 3.1^c^	222.3 ± 1.3^c^
P + EO_1.25_	17.4 ± 0.7^d^	15.2 ± 0.7^d^	238.0 ± 1.1^b^	244.4 ± 1.7^b^	222.8 ± 2.0^b^	235.4 ± 2.7^b^
P + EO_1.5_	16.2 ± 1.1^e^	14.9 ± 0.4^d^	246.6 ± 3.4^a^	250.6 ± 2.2^a^	235.4 ± 3.2^a^	249.1 ± 1.8^a^

Values followed by the same letter are not significantly different according to Duncan’s multiple range test (*P* ≤ 0.05), each value represents the mean of 3 replicates ± SD.

**MDA: malondialdehyde, TPC: total phenolic content, TFC: Total flavonoid content, C: untreated control, P: infected with *F. solani*, P + F: infected with *F. solani* and treated with chemical fungicide (Rhizolex-T), EO_1.0_, EO_1.25_, EO_1.5_: treated with lavender EO at 1.0, 1.25, and 1.5% respectively, P + EO_1.0_, P + EO_1.25_, P + EO_1.5_: infected with *F. solani* and treated with lavender EO at 1.0, 1.25, and 1.5%, respectively, and dap: days after planting.

## Discussion

The present work aimed to evaluate the effect of lavender EO in regard to their antifungal activity against *F. solani* in vitro, and their resistance-inducing activity against *Fusarium* damping-off in sorghum, especially on *SbWRKY* TFs. In vitro, the results indicated that lavender EO possesses concentration dependent antifungal activity against *F. solani*. This result is in agreement with findings reported by Bahmani and Schmidt^23^, and Behmanesh et al.^22^. Antifungal activity of EOs and extracts from different medicinal plants, including lavender EO, has been reported by many researchers^8,9^. The chemical composition of medicinal plants comprises various bioactive phytochemicals such as coumarins, flavonoids, terpenes, anthocyanins, and tannins, which may contribute to the fungitoxic activity. Different mechanisms have been described in this concern including interfering with permeability and integrity of fungal cell walls and plasma membranes, suppression of metabolic enzymes, and DNA damage^24^. GC–MS analysis of lavender EO showed existence of some bioactive constituents with a known antifungal background including linalool as the main bioactive component, in addition to linalyl anthranilate, α-terpineol, 1,8-cineole (eucalyptol), α-Pinene, and limonene. Most of the antifungal activity of lavender EO is attributed to linalool, simply because it is the most abundant bioactive component. Recent researchers have reported a potent antifungal activity for linalool^25^. It’s fungitoxic effect can be explained in the light of interference with cell wall biosynthesis and disrupting permeability of plasmalemma^26^. In addition, α-terpineol has been reported also as a potent antifungal agent and its antimycotic effect has been suggested to be due to its activity on cytoplasmic degeneration and hyphal distortions^27^. These antifungal effects were observed in our TEM observations. In this regard, the TEM observations revealed many abnormalities in cellular ultrastructure of *F. solani* treated with lavender EO such as thickening of cell wall and plasmalemma, indicating that multi-mechanisms contributed to the observed antagonistic behavior, that are compatible with a result loss of permeability. Thickening of the cell wall and plasmalemma leads to restriction of the cellular exchange of ions and molecules with the surrounding medium, which finally results in cell death^8^. In addition, another antifungal mechanism was observed by TEM, which is cytoplasmic coagulation. This effect is correlated with the impairment of the plasmalemma, followed by condensation and coagulation of the cytoplasm and finally cell death^28^. Absence of lipid globules in the treated *F. solani* hyphae was also observed with TEM. Lipid droplets play important roles in the fungal cell as energy reserves, preventing lipotoxicity, and regulating some physiological processes^29^. Absence of lipid globules reveals that the fungal cell is suffering stress conditions.

At the molecular level, twelve genes including three *SbWRKY* TFs, *JERF3* and eight defense-related genes, representing SA-, JA- and ET-signaling pathways, were selected in this study as pathway reporter genes. Transcriptional expression levels of these genes were investigated in sorghum shoot in response to application of lavender EO and/or infection with *F. solani* at 3 and 6 dpe. The obtained results demonstrated that *SbWRKY1* was the highest expressed gene followed by *JERF3*, which suggest probable primary role(s) in the plant resistance in response to these treatments. Plants are subjected to multiple environmental stresses, including pathogenic fungi, and energetically respond to these challenges to survive. In order to overcome the stresses encountered, plants initiate transcriptional cascades through cellular signaling pathways. These pathways interact in coordination with each other via signaling molecules leading to stimulation of the defensive-gene-regulatory networks^30^. Transcription factors, such as *WRKY* proteins, play important roles in synchronously organizing the transcription-regulatory-networks enhancing the plant responses against biotic and abiotic stresses^16^. *WRKY* TFs bind to W-boxes found in the stress-inducible promoters of many defense-related genes in plants. The W-boxes exist in clusters, suggesting coordinated interactions of several *WRKY* TFs^31^. In this regard, *WRKY1* TF has been reported as a key element mediating induced resistance against infection with *Alternaria solani* in wild tomato (*Solanum arcanum*)^32^. *WRKY1* regulates SA-signaling pathway via interaction with *NPR1* gene (Natriuretic Peptide Receptor 1), which functions as a master regulator in the orchestration of the plant-defense-responses, controlling expression of more than 2000 defense-related genes^33,34^. *JERF3*, which functions as a key element of ET/JA-signaling pathways, activates multiple defense responses via binding to the GCC box located in the promoters of some defense-related genes^35^. In this regard, Zhang et al*.*^36^ reported the involvement of *ERF3* in triggering an array of defense responses against *Blumeria graminis* in wheat at early stages via the SA-signaling pathway, and against *F. graminearum* or *Rhizoctonia cerealis* at late stages via ET/JA-signaling pathways. In this study, one of the most interesting results obtained by the hierarchical clustering analysis is the single clustering of *SbWRKY45* away from the other studied genes revealing its unique behavior. This result is in agreement with that obtained by Shimono et al.^37^ who reported the vital role of *OsWRKY45* in triggering plant resistance against the blast fungus (*Magnaporthe grisea*) on rice. The same concept was reported by Qiu and Yu^38^ against *Pyricularia oryzae* and *Xanthomonas oryzae* on rice, and in *Arabidopsis*. The *WRKY45-*induced resistance included overexpression of some *PR* genes, particularly, *PR1* and *PR2* (markers of systemic acquired resistance). In addition, he confirmed the mediation of *OsWRKY45* to SA-signaling pathway. Likewise, *WRKY19* has been also reported to be involved in induction of plant resistance against powdery mildew of barley^39^. It is known that induction of SA-signaling pathway leads to overexpression of the pathogenesis-related (PR) genes *PR1*, *PR2*, and *PR5*, while, triggering JA-signaling pathway induces *PR3*, *PR4*, and *PR12* genes^40^. In this regard, data obtained in this study revealed overexpression of *PR1* (antifungal), *PR2* (*β*-1,3-glucanase), and *PR5* (Thaumatin-like protein) which are SA-responsive defense genes. This result is in accordance with the reported overexpression of *WRKY* genes. PR1 proteins are highly abundant in plants during biotic- and abiotic-stress responses and have been widely used as a defense marker. Unlike PR2 and PR5 proteins, which have known antifungal enzymatic activities, the antifungal mechanism of PR1 proteins remains unclear. However, recent studies have suggested multiple roles of PR1 proteins including sterol-binding activity, hypersensitivity response (cell death), and harboring an embedded defense-signaling peptide (CAP-Derived Peptide 1)^41^. *PR2* encodes the lytic enzyme *β*-1,3-glucanase, which hydrolyz *β*‐1,3‐glycosidic bond in the 1,3-glucan molecules, degrading the cell walls of attacking phytopathogenic fungi^42^. *PR5* encodes antifungal protein which exhibits fungal-cell-wall-lytic activity (glucan binding and glucanase activities), xylanase inhibitor activity, and pathogen recognition (binding with the pathogen cell surface)^43^. The two-genes-clustering (*PR2* and *PR5*) with similar patterns obtained in this study can be explained in the light of their shared glucanase activities and the same SA-signaling pathway. Overexpression of these PR genes implicate a role for SA-signaling in sorghum resistance against *F. solani*. Data from the quantitative Real-Time PCR (qRT-PCR) obtained in this study revealed overexpression of *PR3* (chitinase 15), and *PR12* (Plant defensin 1), which are JA-responsive defense genes. *PR3* encodes the antifungal enzyme chitinase, which catalyze hydrolysis of *β*-1,4 bonds between N-acetylglucosamine subunits of chitin molecules, the main constituent of the fungal cell wall. PR1 and PR3 proteins synergistically inhibit the fungal growth as a plant defense response^42^. Clustering of *PR1* and *PR3* observed in this study is in accordance with the synergism reported between the two proteins in the literature. *PR12* encodes antifungal and cytotoxic proteins, which have significant roles in plant resistance against wide range of phytopathogenic fungi^44^. Overexpression of these *PR* genes revealed the participation of the JA-signaling pathway in sorghum resistance against *F. solani*. *PAL1* is the key gene in the phenylpropanoid pathway regulating biosynthesis of an array of antifungal polyphenolic compounds in plant including flavonoids, lignins, and chlorogenic acid^45^. *GST1* encodes the antioxidant-defense enzyme, which involved in the detoxification function against xenobiotics through binding with glutathione^46^. In addition to *PR* genes, *PAL1* and *GST1* are also defense genes, which regulated by *WRKY* transcription factors. The overexpression of all studied genes was supported with the elevated activities of the estimated antioxidant enzymes and total phenol content explaining the synergistic effect of lavender EO and infection with *F. solani* in triggering the sorghum resistance.

## Materials and methods

### Fungal inoculum, sorghum cultivar, and lavender shrubs

A virulent isolate of the fungus *F. solani* (GenBank accession no.: KJ831188), isolated from sorghum seedling showing damping-off symptoms, was used in this study. The fungal isolate was maintained on potato dextrose agar (PDA) slants and kept at 4 °C until use. For inoculum preparation, fungal spores from 7-days-old PDA cultures of *F. solani* were harvested using sterile water and the spore suspension was adjusted at 1 × 10^6^ spore mL^−1^. Sorghum grains cv. Giza 15, obtained from the Central Administration for Seed Certification, Egypt, were used in the greenhouse experiment. The used lavender shrubs were originally cultivated in the Experimental Nursery of the Medicinal and Aromatic Plants Research Department, Horticultural Research Institute, Agriculture Research Center, Giza, Egypt. The shrubs were harvested in July 2020, identified by Prof. Ibrahim A. Mashaly, and deposited at the Herbarium of Faculty of Science, Mansoura University, Mansoura, Egypt under the deposition number (Mans 0303075019).

### Essential oil extraction

Lavender EO was extracted from 200 g of air-dried lavender flowers via hydro-distillation for 60 min using Clevenger apparatus as described by Zheljazkov et al.^47^. The EO was then filtered and stored in dark bottle at 4 °C until use.

### Screening for antifungal activity of lavender EO in vitro

Antifungal activity of lavender EO was assessed against mycelial growth of *F. solani* in vitro using agar plate technique. PDA plates supplemented with lavender EO at final concentrations of 0.5, 0.75, 1.0, 1.25, and 1.5% were used. The tested concentrations were prepared by adding suitable volumes of EO to 100 mL Erlenmeyer flasks containing melted PDA medium before solidification and 0.5% Tween-80. PDA plates supplemented with a synthetic fungicide (nystatin) at 50 µg/mL were used as positive control. Untreated PDA plates were used as negative control. The PDA plates were inoculated in the centers with 7-mm-diameter discs taken from active margins of 7-days-old culture of *F. solani*. For each treatment, three plates were used. All plates were incubated in dark at 25 ± 2 °C until full fungal growth was obtained in the control plate. Diameter of the fungal colony in each plate was measured and the reduction percentage in mycelial growth was calculated as follows:

$$\text{Reduction} \; \text{percentage }\left({\%}\right)=\frac{\mathrm{C}-\mathrm{T}}{\mathrm{C}}\times 100$$

where C = colony diameter of the control plate, and T = colony diameter of the treated plate.

### SEM

To investigate effects of lavender EO on morphology of *F. solani* using SEM, one block from *F. solani* culture on PDA plate and another one block from *F. solani* culture treated with lavender EO were separately processed using tissue processor (Leica Biosystems, Inc.). The two blocks were fixed using osmium oxide, dehydrated using ethanol, and dried using a critical point drier (EMS 850), and then coated with gold using a sputter coater (EMS 550). Morphology of the fungal structures was observed using SEM (JEOL JSM-6510LV).

### TEM

To investigate effects of lavender EO on ultrastructure of *F. solani* using TEM, samples of the treated plates were fixed using 3% glutaraldehyde in phosphate buffer at pH 6.8, followed with 1% osmium tetroxide, then dehydrated in a gradual ethanol series as described by Alberto et al*.*^48^*.* The dehydrated specimens were embedded in plastic epoxy resin and cut to ultra-thin sections using Reichert ultramicrotome, and stained with uranyl acetate followed by lead citrate. The sections were examined using JEOL-TEM (model JEM-1230).

### GC–MS

Chemical constituents of lavender EO were identified using a GCMS-QP2010 system (Shimadzu, Japan). The EO sample was injected at flow rate of 1.5 mL min^−1^ via a DB-5 column (60 m × 0.25 mm, 0.25 μm thick) using helium as a carrier at 300 °C. The ion source temperature was 210 °C, while the interface temperature was 300 °C, at an ionization voltage of 70 eV. Retention time and mass spectra were used to identify the EO composition using the NIST11library (Gaithersburg, USA).

### Greenhouse experiment

Plastic pots (15 cm diameter) filled with sterile sandy-clay soil (1:2 v/v) were used. For soil infestation, the spore suspension of *F. solani* (1 × 10^6^ spore mL^−1^) was mixed with upper layer soil of the pots at the rate of 2% (v/v) ten days before planting. Sorghum grains were soaked separately in lavender EO at different concentrations (1, 1.25, and 1.5%) for 2 h, then air-dried before planting. For positive control, sorghum grains were treated with the chemical fungicide Rhizolex-T as seed dressing at the recommended dose of 3 g kg^−1^ grains. For each treatment, ten sorghum grains were sown in each pot. Pots planted with surface-sterilized sorghum grains were used as a control treatment. Four replicates were used for each treatment. All pots were regularly irrigated as required, arranged in a complete randomized design, and kept under greenhouse conditions (27/21 °C day/night temperature, 65% humidity). The applied treatments were designated as follows: C: untreated control, EO_1.0,_ EO_1.25,_ EO_1.5_: treated with lavender EO at 1.0, 1.25, and 1.5% respectively, P: infected with *F. solani*, P + EO_1.0_, P + EO_1.25_, and P + EO_1.5_: infected with *F. solani* and treated with lavender EO at 1.0, 1.25, and 1.5% respectively, and P + F: infected with *F. solani* and treated with chemical fungicide. All pots were evaluated for damping-off incidence 45-days after planting (dap). Percentages of seed rot, pre- and post-emergence damping-off were recorded.

### Expression analysis of the defense-related genes

#### Total RNA extraction and cDNA synthesis

Total RNA was extracted from fresh sorghum shoot at 3 and 6 days post seedling emergence (dpe) using RNeasy Mini Kit (Qiagen, Germany) according to the manufacturer’s instructions. The extracted RNA was incubated with DNase for 1 h at 37 °C and quantified using a NanoDrop 1000 spectrophotometer (Thermo Scientific, USA).

For cDNA synthesis, RT-PCR kit (Qiagen, Germany) was used according to the manufacturer’s instructions. The reaction mixture (20µL) contained 2.5 µL dNTPs (2.5 mM), 5 µL 5×-buffer with MgCl_2_, 4 µL oligo (dT) primer (20 pmol µL^−1^), 0.2 µL reverse transcriptase enzyme (Omniscript RT, Qiagen, Germany) and 2 µL RNA. PCR amplification was performed using a thermal cycler (Promega, Germany), at 42 °C for 2 h and 65 °C for 20 min, the cDNA was then stored at − 20 °C until used.

#### qRT-PCR

The reaction mixture included 1 µL of template, 12.5 µL of SYBR Green Master Mix (Bioline, Germany), 1 µL of forward primer, 1 µL of reverse primer, and sterile RNase free water for a total volume of 20 µL. A *β*-actine gene was used as a reference gene. Sequences of used primers are presented in Table 7. The real time PCR program was performed using a Rotor-Gene-6000-system (Qiagene, USA) as follows: one cycle at 95 °C for 10 min, 40 cycles (95 °C for 20 s, 58 °C for 25 s and 72 °C for 30 s). For each sample, three biological and three technical replicates were performed. The comparative CT method (2^−ΔΔCT^) was used to analyze the relative mRNA expression levels according to Livak and Schmittgen^49^.

**Table 7:** Sequences of primer used in this study.

Primer	Abbrev.	Sequence (5′–3′)
*WRKY* transcription factor 1	*SbWRKY1*-F	CGTGCAGCAGCAAAGCAA
	*SbWRKY1*-R	GTCGCAGGTATGCTCGTTGA
*WRKY* transcription factor 19	*SbWRKY19*-F	AATGTCCCTCTGGCGAACTC
	*SbWRKY19*-R	CAGTACACCCAAGGCTCCAT
*WRKY* transcription factor 45	*SbWRKY45*-F	CTCTGGAGACGGAGCTACAC
	*SbWRKY45*-R	CCACCATCTCCGTGTACTGG
Jasmonate and ethylene-response factor 3	*JERF3*-F	GCCATTTGCCTTCTCTGCTTC
	*JERF3*-R	GCAGCAGCATCCTTGTCTGA
Glutathione S-transferase 1	*GST1*-F	CGGTGACTTGTACCTCTTCGAATC
	*GST1*-R	ATCCACCATTGCTGCCTCC
Pathogenesis-related 1	*PR1*-F	TGGACCCTGGAGATTCCGT
	*PR1*-R	GTCGACTCCACCTTCACCAC
Thaumatin-like protein	*PR5*-F	AAATATCTCCAGTATTCACATTC
	*PR5*-R	AAGTCTGTGGCCATAACAGCAA
Phenylalanine ammonia-lyase 1	*PAL1*-F	TCGCAATCGCAAACATC
	*PAL1*-R	TGCCCTTGAACCCGTAGTC
Chitinase 15	*PR3*-F	GGYGGYTGGAATGARGG
	*PR3*-R	GAYTTRGATTGGAATAYCC
*β*-1,3-glucanase	*PR2*-F	CCGATAACCATGGCTTCTTCTTCTCTGCAGTC
	*PR2*-R	TATCATCCTAGGTTACAACCGAAGCTTGATGATGCAAAG
Plant defensin 1	*PR12*-F	CACAGAAGTTGTGCGAGAGG
	*PR12*-R	GCAAGATCCATGTCGTGCTT
Antifungal proteins	*AFPRT*-F	GTCGTCTTCTGCCCATGATT
	*AFPRT*-R	ACGTGGAGCATGGTGTATCA
*β*-Actin	*β*-Actin-F	GTGGGCCGCTCTAGGCACCAA
	*β*-Actin-R	CTCTTTGATGTCACGCACGATTTC

#### Evaluation of plant growth

For each treatment, three plants were carefully uprooted 30 and 45 dap, washed with tap water to remove soil particles, and evaluated for plant height, shoot and root dry weights. Dry weights were measured after the samples oven-dried at 80 °C for 48 h.

### Biochemical analyses

#### Preparation of crude plant extract

Samples of plant roots (2 g) were ground and homogenized in 5 mL of 100 mM phosphate buffer (pH 7). The homogenate was centrifuged at 15,000 rpm for 20 min, then the supernatant was collected and used as crude extract for next enzyme assays and biochemical analyses. The protein content was estimated for the assayed enzymes according to Bradford^50^.

#### Assay of enzymes activities

Activity of CAT was determined according to Aebi^51^. Activity of SOD was determined according to Misra and Fridovich^52^. Activity of ascorbate peroxidase enzyme (APX) was determined according to Nakano and Asada^53^. Activity of PPO was determined according to Duan et al.^54^.

### Lipid peroxidation, and total phenolic and flavonoid contents

Lipid peroxidation was measured as described by Heath and Packer^55^. Total phenolic content was estimated according to Slinkard and Singleton^56^. Total flavonoid content was determined as described by Wang et al.^57^.

### Statistical analyses

Statistical significances were analyzed using the software CoStat (version 6.4). Comparisons between the means were performed using Duncan’s multiple range test^58^ at *P* ≤ 0.05. The hierarchical clustering analysis was performed using BioVinci Software (Bioturing, San Diego, CA, USA) (Supplementary Figure 1).

### Ethics declaration

Authors confirm that all the methods and experiments were carried out in accordance with relevant guidelines and regulations.

## Supplementary Information

Supplementary Figure 1.
