# Supplementary material for: Essential oil from Lavandula angustifolia elicits expression of three SbWRKY transcription factors and defense-related genes against sorghum damping-off
Source: Sci Rep. 2022 Jan 17;12:857. doi: 10.1038/s41598-022-04903-x (PMC8763899; doi:10.1038/s41598-022-04903-x)
Supplement: Supplementary file 1 — Supplementary Figure 1. [file 41598_2022_4903_MOESM1_ESM.docx]

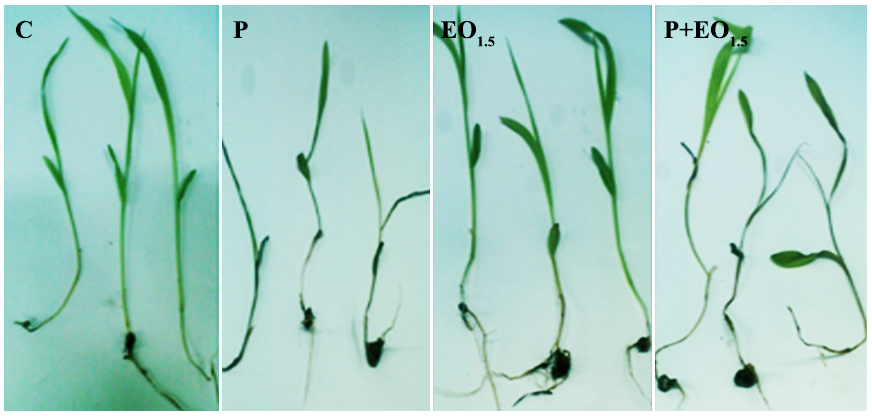


**Fig.** **x.** A photograph showing the disease symptoms on sorghum seedlings infected with *Fusarium solani* and/or treated with lavender essential oil at 1.5%. Where, C: untreated control, P: infected with *F. solani*, EO_1.5_: treated with lavender essential oil at 1.5%, and P+EO_1.5_: infected with *F. solani* and treated with lavender essential oil at 1.5%.
